# Urinary nephrin - An emerging novel biomarker in early detection of diabetic nephropathy: A review

**DOI:** 10.6026/973206300213123

**Published:** 2025-09-30

**Authors:** Ramagiri Sateesh, Kurpad Nagaraj Shashidhar

**Affiliations:** 1 Department of Biochemistry, Sri Devaraj Urs Medical College, Sri Devaraj Urs Academy of Higher Education and Research, Tamaka, Kolar, Karnataka, India

**Keywords:** Diabetes mellitus, diabetic nephropathy, urinary nephrin and biomarker

## Abstract

Diabetic nephropathy is a microvascular complication of uncontrolled diabetes mellitus. Diabetic nephropathy is characterized by persistent albuminuria, hypertension and a progressive decline in the glomerular filtration rate. Early detection of diabetic nephropathy is crucial for the management of complications. Urinary Nephrin is an emerging biomarker that has been implicated in early detection of diabetic nephropathy. Nephrin is a podocyte-specific protein that maintains glomerular filtration barrier integrity. Alterations in Nephrin expression and urinary excretion are signs of renal impairment in diabetic nephropathy. Therefore, it is of interest to establish a biological reference interval and standardize the assay method. Hence, an update on this emerging biomarker is relevant.

## Background:

Diabetes Mellitus (DM) is a non-communicable disease increasing globally. International Diabetes Federation (IDF) documented around 537 million people with diabetes in 2021, exponentially increasing to 643 million by 2030 and 783 million by 2045. Since India has a diverse population with a mix of hereditary, lifestyle, dietary, rural and urban with an aging population, IDF findings also holds good for the Indian population [1]. Diabetic Nephropathy (DN) is the most common microvascular complication of DM, resulting in End Stage Renal Disease (ESRD) or renal failure. Evidence of DN clinically is substantiated by excretion of microalbumin in urine, ranging from 30-300 mg/day. Microalbuminuria is a sequelae of destruction of capillary blood vessels of kidneys, subsequent to uncontrolled, chronic DM and hypertension [2]. There is a felt need for a biomarker for early detection of Diabetes and its complications. Diabetic Nephropathy manifests as distinct pathological, functional and structural alterations in the kidneys [3]. Structural alterations of kidneys include thickening of glomerular basement membrane, expansion of mesangial matrix and ultimately sclerosis or scarring of glomeruli, leading to impairment of kidneys to filter blood effectively, resulting in leakage of protein into urine and a decline in renal function [4]. Glomerular basement membrane (GBM) is a gel-like extracellular network within the glomerulus with 300-350 nm width. It is an essential structural element of glomerular capillary wall and directly affects the barrier's properties. GBM supports glomerular endothelial cells or inner capillary wall and visceral glomerular epithelial cells, termed as podocytes, which lies on outer surface of capillary wall [5].

Podocytes are specialized epithelial cells which adhere to the surface of the glomerular basement membrane, serving a pivotal role in upholding the structural integrity of glomerular filtration barrier [6]. Elevated intracellular glucose levels induce cellular and molecular events in podocytes Viz, Increased production of reactive oxygen species (ROS), formation of advanced glycation end products (AGEs), alterations of polyols and hexosamines levels, activation of protein kinase C (PKC), elevated levels of cytokines and growth factors, abnormal notch signaling, stimulation of the renal renin-angiotensin system (RAS). Abnormal molecular changes mediate functional and morphological alterations of podocytes, directly or indirectly leading to podocyte hypertrophy, foot process effacement and epithelial-mesenchymal transition (EMT), podocyte detachment, autophagy and podocyte apoptosis. Pathological changes in podocytes lead to progressive dysfunction, disruption of the kidney's filtration capacity and progression to DN [7, 8]. Podocytes have long cylindrical interdigitated cytosolic projections termed as foot processes, interconnected by a highly specialized cell-cell junction with uniform thickness and an isoporous zipper-like structure called slit diaphragm [9]. Slit diaphragm(SD) is a multiprotein complex, plays a crucial role in blood filtration and signal transduction within podocytes. Normal physiological function of the SD depends on the coordinated activity of key structural and signaling proteins, such as Nephrin, Podocalyxin, P-cadherin, NEPH1, NEPH2, Podocin, CD2-associated protein (CD2AP) and FAT1. Nephrin plays a vital role in the maintenance of SD integrity and signal transduction [10]. Podocyte injury and SD-related molecules could be used as early biomarkers for DN. Proteinuria is a hallmark of development of DN. Microalbumin is clinically considered as a gold standard and a traditional marker for detection of nephropathy in patients with DM. However, proteinuria tests have limited sensitivity with confounding factors viz, urinary tract infection, cardiovascular disease, periodontitis, hepatitis, colitis and high altitude hypoxia outweighs its utility as a biomarker [11, 12]. In recent years, urinary Nephrin has emerged as a promising biomarker for early detection and monitoring of DN. This review offers a comprehensive overview of the urinary Nephrin's role in DN, highlighting its potential as a diagnostic and prognostic marker. Nephrin, a transmembrane protein was first recognized as a key element of extracellular portion of SD of podocyte. Nephrin is a product of the NPHS1 gene, expressed in glomerulus; whose expression was identified in 1998 by Kestila and colleagues in congenital Nephrotic syndrome of Finnish population [13]. Therefore, it is of interest to review Urinary nephrin as an emerging biomarker in early detection of diabetic nephropathy.

## Molecular structure of nephrin:

Nephrin consists of 1,241 amino acids; it belongs to immunoglobulin superfamily with a molecular weight of 135 kDa without post-translational modifications [13]. Mature form of nephrin has a molecular weight of 180-200 kDa [14]. Nephrin is coded by NPHS1 gene, comprising of 29 exons, covering a distance of 26 kilobases within the chromosomal locus 19q13.1 [15]. Nephrin possess an extra and intracellular domain. Extra cellular domain consists eight Ig-like modules could contributing 28 nm length and one fibronectin type III-like module adding more length to protein. A single molecule could extend through most of the width of the SD. Nephrin is able to expand across the width of the slit diaphragm, in the range of 35 to 45 nanometers. Intracellular domain in Nephrin has no significant homology with substantial similarity to known proteins. Despite nephrin contains nine tyrosine residues, only few of them can undergo phosphorylation by nephrin ligand binding and speculated to have a role in podocyte cell adhesion and signal transduction [13, 16]. Each Ig-like domain of Nephrin contains two cysteine residues, which can form disulfide bridges between Nephrin molecules or with other proteins [16].

## Location of nephrin in the kidney:

Nephrin is located in the filtration regions of the podocyte foot processes within the glomerulus of the kidney. Immunoelectron microscopical studies revealed that nephrin is located in the podocyte slit diaphragm region. Nephrin molecules projecting from two neighboring foot processes are expected to engage in interactions within the filtration slit through homophilic interactions [16]. Expression of Nephrin mRNA was initially observed within the late S-shaped bodies in the process of renal organogenesis [17]. Nephrin mRNA expression was not observed outside of kidney glomerulus in normal or NPHS1 mutated children. Nephrin expression is also observed in porcine [18]. Further, expression of Nephrin is also observed in Zebrafish and medaka glomerulus along with glomerular basement membrane but in a different pattern. Nephrin needs to be integrated to the membrane before the formation of the SD and later moves to the proper site to form the SD [19, 20]. Nephrin is absent in the SD of birds. Birds have larger and thick SD as compared with mammalian glomeruli and do not contain a coding sequence for Nephrin [21, 22]. Birds excrete nitrogen predominantly as uric acid, which has limited solubility in aqueous solutions and requires a significant amount of protein to be maintained in a colloidal suspension in the urine. Due to absence of Nephrin, proteins could pass the glomerular filtration barrier. Indirectly emphasizing the significance of Nephrin in mammals [23].

## Regulation of Nephrin expression:

Maintaining nephrin at the slit diaphragm by either increasing its expression or inhibiting its degradation would be beneficial to proper function of SD in glomerulus [24]. Epigenetic alteration such as DNA methylation and histone acetylation lead to decreased nephrin expression induces disruption of the SD and cause proteinuria [25, 26]. After transcription, various RNA-binding proteins and microRNAs can regulate nephrin expression by modulating mRNA stability and translation efficiency. MicroRNA miR-30 regulates nephrin expression by targeting its mRNA for degradation [27]. Alteration in nephrin expression could play a role in the pathogenesis of Ang II-induced podocyte apoptosis [28].

## Role of Nephrin in slit diaphragm:

In addition to serving as a physical barrier, Nephrin plays a role in signaling pathways that preserve SD turnover, cytoskeletal organisation, calcium mechanosensing and podocyte polarity by phosphorylation of various tyrosine and threonine residues found in specific binding motifs within its cytoplasmic region, assisted by a vast network of cytoplasmic binding partners such as CD2AP, Nck, Podocin, Zonula Occludense-1 [29].

## Nephrin in glomerular diseases:

Mutations in the genetic sequence responsible for encoding the Nephrin (NPHS1) have been identified as causative factors in a spectrum of renal disorders, resulting in a myriad of pathological conditions affecting the functionality and structure of the kidneys. These genetic variations can give rise to a diverse array of kidney diseases, encompassing a range of manifestations and clinical presentations that impact renal function and overall health.

## Nephrin in congenital nephrotic syndrome of Finnish type:

Congenital nephrotic syndrome of Finnish type (CNF) is an autosomal recessive disease resulting of mutations in the NPHS1 gene. Patients with nephrotic syndrome die during the first two years of life as the condition advances quickly after birth. Frequency of CNF is 1:10000 births, two mutations, named Fin major and Fin minor, were found in over 90% of CNF patients. Fin major mutation involves 2 bp deletions (CT) in exon 2, which causes a frame shift and formation of a stop codon at the end of exon 2. This mutation leads to a complete lack of Nephrin protein. Fin minor is a nonsense mutation (C→T) in exon 26, leading to a truncated nephrin protein with 1109 residues [13, 15]. Predominant clinical presentation of CNF is characterized by significant proteinuria, which initiates during fetal development, accompanied by hypoproteinemia and edema. Additional atypical observations consist of premature birth and an enlarged placenta. Patients harboring Fin major and Fin minor genetic mutations exhibit comparable clinical manifestations. Currently, pediatric individuals diagnosed with CNF undergo treatment involving bilateral nephrectomy and dialysis, subsequently followed by renal transplantation [30]. Recurrence of CNF occurred in 20%-25% of the patients with post renal transplantation [31, 32]. Recurrence of NS in CNF children is limited to patients with a Fin major/Fin major homozygous genotype and in patients with significant high levels of serum anti-Nephrin antibodies, suggesting potential autoimmune reaction against Nephrin [33].

## Primary nephrotic syndrome:

Down-regulation of nephrin expression and its altered localization are common findings in various human glomerular diseases and animal models. These changes have been observed in both human patients and experimental animal studies [34]. Doublier *et al.*, observed in an immunofluorescence microscopy study, loss of staining for nephrin and podocyte-staining pattern shifted to a granular pattern in patients with primary nephrotic syndrome [35]. Expression of nephrin is altered in patients with minimal change nephrotic syndrome (MCNS). Granular pattern of nephrin was observed in renal biopsy samples of MCNS patients. Granularization indicates degree of effacement of foot process in podocytes [36]. Nawata *et al.* reported that, reduced Nephrin expression in primary membranous nephropathy by immunohistochemistry (IHC) and quantitative RT-PCR studies [37].

## Diabetic nephropathy:

Humans with diabetic nephropathy, proteinuria may be closely associated with the onset and/or progression of low nephrin mRNA expression or loss of Nephrin at SD. Low mRNA expression of nephrin can lead to alterations in the structure and function of the kidney's filtration barrier, contributing to protein leakage into the urine [38]. Podocyte damage is a pivotal factor in the development of proteinuria in DN. Hence, podocyte-related molecules could be early biomarkers for DN.

## Urinary nephrin as a biomarker in diabetic nephropathy:

Alterations in nephrin expression and its urinary excretion have been observed in early stages of DN. Urinary nephrin levels have been found to be higher in individuals with DN, especially in those who had overt proteinuria. These findings highlight the potential of urinary nephrin as a more sensitive biomarker of early glomerular dysfunction and disease severity in DN than traditional albuminuria [39]. Kondapi *et al.* found a significant difference between Urinary Nephrin and Microalbumin in T2DM patients. Hyperglycemia induces alterations in the expression and phosphorylation of nephrin, thereby playing a role in the development of renal injury. In individuals with T2Dm, nephrin has been identified as a more sensitive indicator of early kidney dysfunction compared to albuminuria [40]. A cross-sectional study found that patient with DN has higher urine nephrin levels than people without nephropathy. This finding raises the possibility of a relationship between elevated urinary nephrin levels and the development of nephropathy in T2DM patients [41]. Study conducted by Belinda *et al.*, suggested urinary nephrin may be a potential biomarker for early prediction of DN [42]. These studies propose estimation of urinary Nephrin cloud be a more sensitive and promising biomarker in early detection of DN.

## Diagnostic and Prognostic utility of urinary Nephrin in DN:

Presence and degree of albuminuria/proteinuria reflects the extent of glomerular damage. Off late, newer biomarkers play a key role in early detection and assess the prognosis of DN. Nephrin levels in urine could be a promising and non-invasive diagnostic marker for the early detection of DN. Quantification of urinary nephrin is a potential marker for identifying kidney damage even before the symptoms appear and enables clinician's timely intervention and management. Elevated urinary nephrin levels have been found to correlate with the severity of albuminuria, indicating a close relationship between nephrin excretion and disease severity. Studies have demonstrated that even in the absence of overt proteinuria, elevated urinary nephrin levels are linked to the assessment of extent of development of DN. Present day research indicates the prognostic significance of urinary nephrin in forecasting the advancement of DN [43, 44-45]. Despite "Hue and cry" on Nephrin as an early marker, methodology and its validation are yet to be understood. Among the various methodologies, "Gold standard" method has to be derived. ELISA, RT-PCR and Western blotting techniques are the popular methodologies for quantification of Nephrin in urine. ELISA has been demonstrated to have an excellent diagnostic accuracy compared to RT-PCR. Since each method has its strengths, opportunities, weaknesses, challenges and limitations, multiple approaches provide a comprehensive understanding of urinary Nephrin [46].

## "Road map" ahead of Nephrin:

Early and timely evaluation with simple diagnostic modalities is beneficial for identifying and managing DN. Studies have elucidated Nephrin as a potential urinary biomarker. Despite its potentiality challenges restrict its application as a biomarker for DN. Standardization of assay methodologies and reference ranges are essential to ensure reproducibility and comparability of results across different studies. Future research may elucidate the mechanisms underlying the dysregulation of urinary nephrin in DN and its potential use as a therapeutic target.

## Nephrin in preeclampsia:

A recent cross-sectional study revealed that preeclamptic pregnancy has higher urinary values compared to normotensive pregnancy. There is no substantial correlation between the severities of preeclampsia with raised urinary nephrin levels. These factors stress upon the futuristic trend in research related to urinary nephrin as a screening and diagnostic marker for preeclampsia [47]. Kostovska *et al.* have observed a weak positive correlation between urinary nephrin levels and gestational age. They observed that there was a good statistical correlation. They also suggested that elevated Urinary nephrin levels could be a useful predictor of preeclampsia in women with a high-risk pregnancy [48]. Nephrin being the molecule of interest for the renal system, off late it has been extrapolated to extra renal systems. To mention well-documented organs, Nephrin has expressed its impact on CNS, CVS, endocrine pancreas, genitourinary, uteroplacental and pregnancy, lymphatic system and dermatological manifestations.

## Extrarenal expression of Nephrin:

Nephrin's expression extends beyond the podocyte within the kidney. It is also detected in the central nervous system (CNS), heart, pancreas, placenta, lymphoid tissue, testis and skin as represented in [49]. This suggests that Nephrin might serve a unique role in various tissues.

## Central nervous system implications:

Nephrin's expression is found in the key regions of the developing mouse brain including fourth ventricle, spinal cord, radial glial cells of the cerebellum, hippocampus and olfactory bulb [50]. Nephrin in matured rodents is found in pons and corpus callosum, as well as in granule cells and Purkinje cells of the cerebellum. An *in vitro* investigation revealed that nephrin was expressed in close proximity to synaptic proteins and interacts with PSD95, Fyn kinase and glutamate receptors. However, the exact role of Nephrin in the brain is yet to be studied and has good research potential [51].

## Cardiovascular Involvement:

In addition to Nephrin's role in podocytes, it is also documented its requirement for cardiovascular development. Nephrin is expressed in the epicardium and coronary vessels during human and mouse embryonic development. Nephrin-deficient embryos have displayed a morphological alteration of epicardial cells with developmental defects and affect coronary vessels following an increased apoptosis, with cardiac fibrosis [52].

## Pancreatic mitigation:

Nephrin expression has been reported in the beta cells of pancreas. With limited documentation of its possibilities of serving as a structural protein in islet microendothelium [53]. Nephrin expression was verified with RT-PCR and by sequencing nephrin from human pancreatic cDNA library. Further, Dual immunofluorescence analysis demonstrated that nephrin is precisely localized within the beta cells of the islets of the pancreas [54]. These findings provide new directions for exploring Nephrin's role in Pancreas. However, further research is needed to elucidate the specific role of nephrin in pancreas.

## Testicular expression:

Expression of nephrin mRNA was first detected in the testes in 1-6 weeks-old mice. In-situ hybridization revealed the expression of nephrin gene in Sertoli cells. Furthermore, immunofluorescent labeling investigations revealed that nephrin was co-localized with anchoring protein ZO-1 in the mouse testis. These findings suggest that nephrin plays a crucial role in the barrier system in the testes [55]. However, there are limited reviews with respect to humans.

## Placental expression:

In human placenta, nephrin mRNA expression was higher in chorion than in villi and amnion. Nephrin gene was found in the villous cytotrophoblast cells and endothelium of intravillous vessels. However, Nephrin's specific role in the placenta and related disorders need to be evaluated [56].

## Lymphoid tissue role:

Nephrin expression can be found in lymphoid tissues, specifically tonsils, adenoids and lymph nodes. Astrom *et al.* observed higher levels of Nephrin mRNA expression in tonsils and adenoids than in thymus or B lymphocytes and monocytes by RT-PCR technique. However, specific role of Neprin in lymphoid tissue is a gap to be addressed [57].

## Dermatological effect:

Nephrin has been documented with a varied physiological role such as cell adhesion, proliferation and migration in human skin. Nephrin was expressed on the margins of re-epithelized epidermis *in vivo* mice as well as human skin wounds [58].

## Role of vitamins in expression of Nephrin:

Studies have demonstrated that all-trans retinoic acid (ATRA) and 1,25-dihydroxyvitamin D3 has an impact on nephrin expression in murine podocytes. Nephrin expression focuses on three receptors viz retinoic acid receptor (RAR), retinoid X receptor (RXR), and vitamin D receptor (VDR). ATRA and vitamin D3 influence nephrin expression through RAR, RXR and VDR receptors, probably via molecular pathways. Understanding the receptor mechanism of action provides insights into how ATRA and vitamin D3 benefit kidney function, with potential implications for therapeutic interventions targeting RAR, RXR and VDR receptors to preserve nephrin expression and mitigate kidney damage [59, 60]. It has been documented the beneficial effects of Vitamin D on nephrin and function of podocytes. Both hereditary and acquired 1,25-vitamin D3 deficiency are associated with decreased nephrin expression and subsequent glomerular damage, leading to increased risk of proteinuria [61]. Further studies are required to elucidate the basic and molecular mechanisms and optimize its therapeutic strategies. Oxidative stress is known to affect nephrin expression and disrupt renal filtration function. Vitamin E as an antioxidant is observed to reduce oxidative stress markers such as renal malondialdehyde (MDA) levels and parallelly increase the activities of superoxide dismutase (SOD). Vitamin E also preserves the nephrin expression and maintains kidney health by counteracting damage caused by oxidative stress parameters [62]. Oxidative stress plays a crucial role in the development and progression of preeclampsia by adversely affecting nephrin expression, shedding and cellular localization, ultimately contributing to kidney dysfunction and proteinuria in affected individuals [63]. Inflammatory cytokines, such as interleukin-1 beta (IL-1β) and tumor necrosis factor-alpha (TNF-α), have been found to decrease the expression of nephrin. This downregulation occurs through the activation of a cellular signaling pathway via phosphatidylinositol-3-kinase (PI3K)/Akt pathway and disrupts the kidney's filtration barrier, leading to proteinuria and renal dysfunction [64].

## Nephrin expression in HIV:

It has been shown by Western blot and immunofluorescence, in human cultured podocytes, that HIV-associated protein Tat makes albumin more permeable by glomerulus. Moreover, in cultivated podocytes, that can rapidly result in the loss and redistribution of nephrin, resulting in increased glomerular permeability and proteinuria, which is consistent with HIV-associated nephropathy [65].

## Calcium regulation by Nephrin:

Recent research suggests that Nephrin may have additional functions beyond its structural role. Nephrin may also play a significant role in the regulation of calcium homeostasis within podocyte. Proper calcium homeostasis is essential for the normal functioning of podocytes, as it affects various cellular processes, including cell signaling, contraction and gene expression [66]. Urinary biomarkers assess glomerular injury, tubular injury, inflammatory and fibrotic pathways. They offer greater potential for early detection, risk assessment and management strategies in diabetic nephropathy. Urinary nephrin is an emerging, sensitive and specific biomarker for podocyte injury. It offers a distinct opportunity for intervention before traditional biomarkers. Integration of nephrin with multi-marker panels and omics-based methods, viz., genomics, transcriptomics, proteomics and metabolomics, enables a comprehensive view of molecular pathways linked with nephrin-related injury. Further, it provides a potential framework for understanding, diagnosis and monitoring the glomerular disease and transform clinical approach to early detection and management of diabetic nephropathy [67, 68-69]. We have tried to explore the potential use of urinary Nephrin as an emerging biomarker for early detection of diabetic nephropathy. We also looked into the multifacetal role of urinary nephrin, such as its significance in early detection of DN, clinical value and significance in glomerular function. Additionally, this article assesses the feasibility of integrating urinary nephrin measurement as a routine clinical practice.

## Conclusion:

Urinary Nephrin represents an emerging and promising biomarker for early detection of DN. Its specificity and sensitivity to glomerular injury, ability to predict disease progression and potential for early intervention make it a valuable tool in the management of DN. Research efforts to validate reference range and standardize urinary nephrin assays are essential to realize its clinical utility and integrate it into routine clinical practice for the early detection and management of DN and also its mitigation in extra-renal tissues.
